# Poisoning by Tobacco

**Published:** 1865-01

**Authors:** 


Poisoning by Tobacco.—Dr. Gallavardin in speaking of the fact communicated to the Academy by Dr. Namias, and which I mentioned in a former letter concerning a smuggler nearly poisoned to death by concealing a quantity of tobacco about his person under his shirt, mentions three similar cases which occurred in 1801, 1844, and 1854. The first was that of a whole squadron of cavalry, the men having covered their bodies with tobacco leaves with a view to smuggle them across the frontiers. Though they were all inveterate smokers, they all experienced headache, vertigo, and sickness. The second case was that of a woman of fifty, who had done the same thing, and was in consequence attacked with nausea and vomiting, hiccough, oppression of the chest, cold perspiration, chilliness, especially at the extremities, and a slow and intermittent pulse. In the third case, tobacco leaves with honey spread over them were applied to the limbs of a patient who was suffering from rheumatism. He experienced symptoms similar to the above, and the same phenomena have been observed where tobacco juice, or the leaf of the plant was applied to chronic eruptions, or upon parts where the skin had come off, or upon ulcers and sores. Hence Dr. Gallavardin concludes that tobacco under such circumstances produces symptoms similar to those it produces when absorbed in other ways. W. N. Cote.

